# Supplementary material for: HIVprotI: an integrated web based platform for prediction and design of HIV proteins inhibitors
Source: J Cheminform. 2018 Mar 9;10:12. doi: 10.1186/s13321-018-0266-y (PMC5845081; doi:10.1186/s13321-018-0266-y)
Supplement: Supplementary file 2 — Additional file 2. Source code of HIVProtI web server. [file 13321_2018_266_MOESM2_ESM.zip › HIVprotI_Source-code/hivprotI/weka-3-6-6/changelogs/CHANGELOG-3-5-6.html]

Weka-3-5-6 Changelog


# Weka-3-5-6 Changelog

Designed for use with Apache Ant.

---

2007-06-01 04:32 fracpete

```
updated version
```

- core/version.txt (1.5)

2007-06-01 04:02 fracpete

```
updated to reflect changes in GenericPropertiesCreator.props file
```

- gui/GenericObjectEditor.props (1.117)

2007-05-31 23:13 fracpete

```
added note to Javadoc of add(Instance) method, that string and relational values are not transferred
```

- core/Instances.html (1.72)

2007-05-31 04:24 fracpete

```
Exceptions were thrown in case of nominal attributes with no labels (e.g., returned by the weka.core.converters.DatabaseLoader), added checks regarding the length of the AttributeStats.nominalCounts array
```

- gui/AttributeVisualizationPanel.html (1.27)

2007-05-31 03:43 fracpete

```
up till now, the loaders were broken regarding String and Relational attributes in the incremental case (the Instances structure they were added to didn't get updated regarding string or relational values). hence an interface change was necessary, i.e., getNextInstances got a parameter, an Instances object that gets updated. All dependend classes had to be updated as well.
```

- gui/explorer/ClassifierPanel.html (1.106)

2007-05-31 03:43 fracpete

```
up till now, the loaders were broken regarding String and Relational attributes in the incremental case (the Instances structure they were added to didn't get updated regarding string or relational values). hence an interface change was necessary, i.e., getNextInstances got a parameter, an Instances object that gets updated. All dependend classes had to be updated as well.
```

- classifiers/Evaluation.html (1.77)
- clusterers/ClusterEvaluation.html (1.40)
- core/converters/AbstractFileLoader.html (1.2)
- core/converters/AbstractLoader.html (1.9)
- core/converters/ArffLoader.html (1.18)
- core/converters/C45Loader.html (1.14)
- core/converters/CSVLoader.html (1.13)
- core/converters/ConverterUtils.html (1.13)
- core/converters/DatabaseLoader.html (1.12)
- core/converters/LibSVMLoader.html (1.3)
- core/converters/Loader.html (1.10)
- core/converters/SerializedInstancesLoader.html (1.13)
- core/converters/TextDirectoryLoader.html (1.2)
- core/converters/XRFFLoader.html (1.3)
- filters/Filter.html (1.38)
- gui/beans/Loader.html (1.16)

2007-05-31 03:42 fracpete

```
added EMPTY_NOMINAL_ATTRIBUTES and EMPTY_NOMINAL_CLASS to the list of capabilities, since the weka.core.converters.DatabaseLoader can generate nominal attributes without any labels (the corresponding capability for the class only for completeness' sake)
```

- core/Capabilities.html (1.20)

2007-05-31 02:56 fracpete

```
fixed Javadoc comment
```

- filters/unsupervised/attribute/RandomProjection.html (1.10)

2007-05-30 04:02 fracpete

```
relational attributes are now filled according to extra parameters (better control over generated data)
```

- core/TestInstances.html (1.9)

2007-05-29 02:54 fracpete

```
added "headless" property, in case the JUnit tests (via "run_tests") need to be run in a headless environment, e.g., via a cronjob
```

- build.xml (1.39)

2007-05-29 01:18 fracpete

```
initial import
```

- associations/FilteredAssociator.html (1.1)
- associations/SingleAssociatorEnhancer.html (1.1)

2007-05-28 21:52 fracpete

```
Date values are no longer output as Doubles but as Date strings again
interestingly enough, the number of seconds seems to differ on Windows (= 986212800000; java 1.5.0_10) and Linux (= 986256000000; java 1.5.0_11) for the same  date (= 2001-04-03)
```

- core/SparseInstance.html (1.20)

2007-05-26 10:42 fracpete

```
removed the SplashScreen for the KnowledgeFlow. somehow didn't start up when run as SDI (MDI worked fine, as well as debugging it - timing issue due to multiple threads?)
```

- gui/Main.html (1.12)

2007-05-25 00:23 fracpete

```
main GUI can be either MDI or SDI driven
window list menu is now sorted
added extension mechanism via MainMenuExtension interface
```

- gui/GenericPropertiesCreator.props (1.16)
- gui/MainMenuExtension.html (1.1)
- gui/arffviewer/ArffViewerMainPanel.html (1.7)

2007-05-24 02:31 fracpete

```
calculated in-bag-error not out-of-bag-error (duh!) - thanks to Mahesh Joshi
```

- classifiers/meta/Bagging.html (1.39)

2007-05-22 23:18 cvs\_rrb

```
BDeu fix
```

- classifiers/bayes/net/search/local/LocalScoreSearchAlgorithm.html (1.7)

2007-05-22 02:57 fracpete

```
fixed typo in globalInfo/Javadoc
```

- filters/supervised/instance/Resample.html (1.11)

2007-05-22 01:56 fracpete

```
fixed copyright
```

- core/neighboursearch/CoverTree.html (1.2)

2007-05-22 01:56 fracpete

```
fixed copyright
```

- core/neighboursearch/covertrees/Stack.html (1.2)

2007-05-21 23:37 cvs\_rrb

```
BDeu score fix
```

- classifiers/bayes/net/search/local/Scoreable.html (1.4)

2007-05-21 23:37 cvs\_rrb

```
BDue score fix
```

- classifiers/bayes/net/estimate/DiscreteEstimatorBayes.html (1.6)

2007-05-17 20:31 fracpete

```
added get-method for obtaining the best options that were found with CV
```

- classifiers/meta/CVParameterSelection.html (1.35)

2007-05-17 04:44 fracpete

```
initial import
```

- filters/unsupervised/attribute/KernelFilter.html (1.1)

2007-05-17 03:34 fracpete

```
Ashraf's modifications regarding nearest neighbor search: BallTree, CoverTree, KDTree, LinearNNSearch
```

- classifiers/lazy/IBk.html (1.40)
- classifiers/lazy/LWL.html (1.21)
- clusterers/XMeans.html (1.23)
- core/DistanceFunction.html (1.6)
- core/EuclideanDistance.html (1.11)
- core/Instance.html (1.25)
- core/KDTree.html (1.10)
- core/LinearNN.html (1.6)
- core/NearestNeighbourSearch.html (1.6)
- core/neighboursearch/BallTree.html (1.1)
- core/neighboursearch/KDTree.html (1.1)
- core/neighboursearch/LinearNNSearch.html (1.1)
- core/neighboursearch/NearestNeighbourSearch.html (1.1)
- core/neighboursearch/PerformanceStats.html (1.1)
- core/neighboursearch/TreePerformanceStats.html (1.1)
- core/neighboursearch/balltrees/BallNode.html (1.1)
- core/neighboursearch/balltrees/BallSplitter.html (1.1)
- gui/GUIEditors.props (1.6)

2007-05-17 03:34 fracpete

```
Ashraf's modifications regarding nearest neighbor search: BallTree, CoverTree, KDTree, LinearNNSearch
```

- core/neighboursearch/balltrees/BallTreeConstructor.html (1.1)
- core/neighboursearch/balltrees/BottomUpConstructor.html (1.1)
- core/neighboursearch/balltrees/MedianDistanceFromArbitraryPoint.html (1.1)
- core/neighboursearch/balltrees/MedianOfWidestDimension.html (1.1)
- core/neighboursearch/balltrees/MiddleOutConstructor.html (1.1)
- core/neighboursearch/balltrees/PointsClosestToFurthestChildren.html (1.1)
- core/neighboursearch/balltrees/TopDownConstructor.html (1.1)
- core/neighboursearch/kdtrees/KDTreeNode.html (1.1)
- core/neighboursearch/kdtrees/KDTreeNodeSplitter.html (1.1)
- core/neighboursearch/kdtrees/KMeansInpiredMethod.html (1.1)
- core/neighboursearch/kdtrees/MedianOfWidestDimension.html (1.1)
- core/neighboursearch/kdtrees/MidPointOfWidestDimension.html (1.1)
- core/neighboursearch/kdtrees/SlidingMidPointOfWidestSide.html (1.1)

2007-05-14 04:07 fracpete

```
Moved I/O for reading an ARFF file from weka.core.Instances to weka.core.converters.ArffLoader.ArffReader. This fixes the problem with the ArffLoader reporting wrong line numbers, due to the use of the Instances.readInstance(Reader) method.
```

- core/Attribute.html (1.44)

2007-05-14 03:39 fracpete

```
fixed Javadoc
```

- gui/GenericPropertiesCreator.html (1.11)

2007-05-14 03:12 fracpete

```
added a method to "sanitize" all the filenames, which are based on the relationname: all the slashes, backslashes and colons are replaced by underscores. Was problematic for filters that had filenames in their options, which in turn had a relation name (under windows) containing backslashes as result. This generated an exception in the ArffSaver used directly after the filter.
```

- gui/beans/Saver.html (1.5)

2007-05-11 03:59 eibe

```
Fixed bug in output of coefficients and intercept: they needed to be multiplied by (J-1)/J, where J is the number of classes. This affected LMT and SimpleLogistic.
```

- classifiers/trees/lmt/LMTNode.html (1.7)
- classifiers/trees/lmt/LogisticBase.html (1.8)

2007-05-11 00:00 fracpete

```
Sebastian Beer's (fixed) contribution, based on:
Ramakrishnan Srikant, Rakesh Agrawal (1996). Mining Sequential Patterns: Generalizations and Performance Improvements.
```

- associations/GeneralizedSequentialPatterns.html (1.1)
- associations/gsp/Element.html (1.1)
- associations/gsp/Sequence.html (1.1)

2007-05-10 23:59 fracpete

```
testing now also data with no class attribute set
```

- associations/CheckAssociator.html (1.5)

2007-05-10 22:44 fracpete

```
creates a copy of the data now in buildAssociations to avoid modification of the original data
```

- associations/Apriori.html (1.28)
- associations/PredictiveApriori.html (1.11)

2007-05-09 04:23 fracpete

```
sets now a default cluster type (uniform/random) if no option available
```

- datagenerators/clusterers/SubspaceClusterDefinition.html (1.4)

2007-05-09 04:23 fracpete

```
relation name is now unquoted when set in setOptions (since it is quoted in getOptions)
```

- datagenerators/DataGenerator.html (1.6)

2007-05-09 04:18 fracpete

```
added "unquote" method (the inverse of "quote")
```

- core/Utils.html (1.57)

2007-05-08 23:21 fracpete

```
fixed tool tips
```

- classifiers/meta/ThresholdSelector.html (1.39)

2007-05-08 23:21 fracpete

```
fixed tool tips
```

- classifiers/bayes/AODE.html (1.17)

2007-05-08 22:22 fracpete

```
Instance-based constructor now also works with SparseInstance
fixed Javadoc
```

- core/AlgVector.html (1.9)

2007-05-08 05:58 fracpete

```
fixed tool tips
```

- datagenerators/ClusterDefinition.html (1.4)

2007-05-08 05:33 fracpete

```
fixed tool tips
```

- attributeSelection/RaceSearch.html (1.24)

2007-05-08 05:26 fracpete

```
fixed tool tips
```

- clusterers/EM.html (1.40)
- clusterers/MakeDensityBasedClusterer.html (1.13)

2007-05-08 05:02 fracpete

```
added default constructor for default setttings
check-methods are now public for use in JUnit tests
```

- core/CheckGOE.html (1.3)

2007-05-08 04:55 fracpete

```
fixed tool tips
```

- filters/unsupervised/attribute/AddNoise.html (1.8)
- filters/unsupervised/attribute/ClusterMembership.html (1.14)
- filters/unsupervised/attribute/MergeTwoValues.html (1.9)
- filters/unsupervised/attribute/PartitionedMultiFilter.html (1.4)
- filters/unsupervised/attribute/PropositionalToMultiInstance.html (1.6)

2007-05-08 00:08 fracpete

```
added \r to list of delimiters
setDelimiters can handle strings \r, \n, \t now and turns them into their character representations
```

- core/tokenizers/CharacterDelimitedTokenizer.html (1.2)

2007-05-07 23:00 fracpete

```
added private method updateExperiment() to update the underlying Experiment object ("add configuration" or "enter configuration" didn't update the experiment object, only the JList model)
```

- gui/experiment/AlgorithmListPanel.html (1.21)

2007-05-07 22:23 fracpete

```
replaced existing copyright notices with correct ones (the university owns the copyright of stuff developed by people working for the university or what students produce during their projects/theses)
```

- classifiers/lazy/KStar.html (1.8)

2007-05-07 21:19 fracpete

```
replaced existing copyright notices with correct ones (the university owns the copyright of stuff developed by people working for the university or what students produce during their projects/theses)
```

- classifiers/lazy/IB1.html (1.19)

2007-05-01 01:24 fracpete

```
replaced existing copyright notices with correct ones (the university owns the copyright of stuff developed by people working for the university or what students produce during their projects/theses)
```

- associations/AprioriItemSet.html (1.5)
- associations/Associator.html (1.9)
- associations/CARuleMiner.html (1.3)
- associations/CaRuleGeneration.html (1.3)
- associations/ItemSet.html (1.12)

2007-05-01 01:24 fracpete

```
replaced existing copyright notices with correct ones (the university owns the copyright of stuff developed by people working for the university or what students produce during their projects/theses)
```

- experiment/Compute.html (1.5)
- experiment/CostSensitiveClassifierSplitEvaluator.html (1.15)
- experiment/CrossValidationResultProducer.html (1.16)
- experiment/DatabaseResultListener.html (1.13)
- experiment/DatabaseResultProducer.html (1.17)
- experiment/DatabaseUtils.html (1.31)
- experiment/Experiment.html (1.26)
- experiment/InstanceQuery.html (1.21)
- experiment/InstancesResultListener.html (1.10)
- experiment/LearningRateResultProducer.html (1.8)
- experiment/OutputZipper.html (1.7)
- experiment/PairedCorrectedTTester.html (1.11)
- experiment/PairedStats.html (1.9)
- experiment/PairedStatsCorrected.html (1.4)
- experiment/PairedTTester.html (1.34)
- experiment/PropertyNode.html (1.6)
- experiment/RandomSplitResultProducer.html (1.19)
- experiment/RegressionSplitEvaluator.html (1.22)
- experiment/RemoteEngine.html (1.9)
- experiment/RemoteExperiment.html (1.15)
- experiment/RemoteExperimentEvent.html (1.6)
- experiment/RemoteExperimentListener.html (1.5)
- experiment/RemoteExperimentSubTask.html (1.8)
- experiment/ResultListener.html (1.7)
- experiment/ResultProducer.html (1.7)
- experiment/SplitEvaluator.html (1.7)
- experiment/Stats.html (1.11)
- experiment/Task.html (1.8)
- experiment/TaskStatusInfo.html (1.4)
- filters/AllFilter.html (1.10)

2007-05-01 01:24 fracpete

```
replaced existing copyright notices with correct ones (the university owns the copyright of stuff developed by people working for the university or what students produce during their projects/theses)
```

- classifiers/bayes/ComplementNaiveBayes.html (1.8)
- classifiers/bayes/NaiveBayes.html (1.21)
- classifiers/bayes/NaiveBayesMultinomialUpdateable.html (1.2)
- classifiers/bayes/NaiveBayesSimple.html (1.19)
- classifiers/bayes/NaiveBayesUpdateable.html (1.9)
- classifiers/bayes/net/ADNode.html (1.6)
- classifiers/bayes/net/BIFReader.html (1.11)
- classifiers/bayes/net/BayesNetGenerator.html (1.12)
- classifiers/bayes/net/ParentSet.html (1.6)
- classifiers/bayes/net/VaryNode.html (1.5)
- classifiers/bayes/net/estimate/BMAEstimator.html (1.7)
- classifiers/bayes/net/estimate/BayesNetEstimator.html (1.3)
- classifiers/bayes/net/estimate/SimpleEstimator.html (1.5)
- classifiers/bayes/net/search/SearchAlgorithm.html (1.7)
- classifiers/bayes/net/search/ci/CISearchAlgorithm.html (1.6)
- classifiers/bayes/net/search/ci/ICSSearchAlgorithm.html (1.7)
- classifiers/bayes/net/search/fixed/FromFile.html (1.7)
- classifiers/bayes/net/search/fixed/NaiveBayes.html (1.5)
- classifiers/bayes/net/search/global/GeneticSearch.html (1.4)
- classifiers/bayes/net/search/global/GlobalScoreSearchAlgorithm.html (1.9)
- classifiers/bayes/net/search/global/HillClimber.html (1.8)
- classifiers/bayes/net/search/global/K2.html (1.6)
- classifiers/bayes/net/search/global/RepeatedHillClimber.html (1.5)
- classifiers/bayes/net/search/global/SimulatedAnnealing.html (1.4)
- classifiers/bayes/net/search/global/TAN.html (1.6)
- classifiers/bayes/net/search/global/TabuSearch.html (1.4)

2007-05-01 01:24 fracpete

```
replaced existing copyright notices with correct ones (the university owns the copyright of stuff developed by people working for the university or what students produce during their projects/theses)
```

- filters/unsupervised/attribute/TimeSeriesTranslate.html (1.8)
- filters/unsupervised/instance/NonSparseToSparse.html (1.4)
- filters/unsupervised/instance/Randomize.html (1.6)
- filters/unsupervised/instance/RemoveFolds.html (1.5)
- filters/unsupervised/instance/RemoveFrequentValues.html (1.5)
- filters/unsupervised/instance/RemoveMisclassified.html (1.7)
- filters/unsupervised/instance/RemovePercentage.html (1.7)
- filters/unsupervised/instance/RemoveRange.html (1.7)
- filters/unsupervised/instance/RemoveWithValues.html (1.12)
- filters/unsupervised/instance/Resample.html (1.9)
- filters/unsupervised/instance/SparseToNonSparse.html (1.5)
- gui/AttributeListPanel.html (1.3)
- gui/AttributeSelectionPanel.html (1.9)
- gui/AttributeSummaryPanel.html (1.11)
- gui/ComponentHelper.html (1.3)
- gui/CostMatrixEditor.html (1.11)
- gui/CustomPanelSupplier.html (1.3)
- gui/DatabaseConnectionDialog.html (1.7)
- gui/ExtensionFileFilter.html (1.7)
- gui/FileEditor.html (1.8)
- gui/GUIChooser.html (1.32)
- gui/GenericArrayEditor.html (1.18)
- gui/GenericObjectEditor.html (1.57)
- gui/HierarchyPropertyParser.html (1.5)

2007-05-01 01:24 fracpete

```
replaced existing copyright notices with correct ones (the university owns the copyright of stuff developed by people working for the university or what students produce during their projects/theses)
```

- classifiers/meta/AdaBoostM1.html (1.37)
- classifiers/meta/AdditiveRegression.html (1.23)
- classifiers/meta/AttributeSelectedClassifier.html (1.25)
- classifiers/meta/ClassificationViaRegression.html (1.26)
- classifiers/meta/CostSensitiveClassifier.html (1.28)
- classifiers/meta/END.html (1.7)
- classifiers/meta/FilteredClassifier.html (1.27)
- classifiers/meta/LogitBoost.html (1.39)
- classifiers/meta/MetaCost.html (1.23)
- classifiers/meta/MultiClassClassifier.html (1.43)
- classifiers/meta/MultiScheme.html (1.24)
- classifiers/meta/OrdinalClassClassifier.html (1.17)
- classifiers/meta/RacedIncrementalLogitBoost.html (1.11)
- classifiers/meta/RandomCommittee.html (1.11)
- classifiers/meta/RandomSubSpace.html (1.3)
- classifiers/meta/RegressionByDiscretization.html (1.35)
- classifiers/meta/Stacking.html (1.31)
- classifiers/meta/StackingC.html (1.13)

2007-05-01 01:24 fracpete

```
replaced existing copyright notices with correct ones (the university owns the copyright of stuff developed by people working for the university or what students produce during their projects/theses)
```

- gui/beans/AbstractTrainingSetProducerBeanInfo.html (1.2)
- gui/beans/Associator.html (1.3)
- gui/beans/AssociatorBeanInfo.html (1.2)
- gui/beans/AssociatorCustomizer.html (1.5)
- gui/beans/AttributeSummarizer.html (1.9)
- gui/beans/AttributeSummarizerBeanInfo.html (1.2)
- gui/beans/BatchClassifierEvent.html (1.6)
- gui/beans/BatchClassifierListener.html (1.2)
- gui/beans/BatchClustererEvent.html (1.3)
- gui/beans/BatchClustererListener.html (1.2)
- gui/beans/BeanCommon.html (1.3)
- gui/beans/BeanConnection.html (1.7)
- gui/beans/BeanInstance.html (1.8)
- gui/beans/BeanVisual.html (1.9)
- gui/beans/ChartEvent.html (1.4)
- gui/beans/ChartListener.html (1.3)
- gui/beans/ClassAssigner.html (1.13)
- gui/beans/ClassAssignerBeanInfo.html (1.2)
- gui/beans/ClassAssignerCustomizer.html (1.7)
- gui/beans/ClassValuePicker.html (1.7)
- gui/beans/ClassValuePickerBeanInfo.html (1.3)
- gui/beans/ClassValuePickerCustomizer.html (1.6)
- gui/beans/Classifier.html (1.25)
- gui/beans/ClassifierBeanInfo.html (1.2)
- gui/beans/ClassifierCustomizer.html (1.11)
- gui/beans/ClassifierPerformanceEvaluator.html (1.15)
- gui/beans/ClassifierPerformanceEvaluatorBeanInfo.html (1.5)
- gui/beans/Clusterer.html (1.5)
- gui/beans/ClustererBeanInfo.html (1.2)
- gui/beans/ClustererCustomizer.html (1.5)
- gui/beans/ClustererPerformanceEvaluator.html (1.3)
- gui/beans/ClustererPerformanceEvaluatorBeanInfo.html (1.2)

2007-05-01 01:24 fracpete

```
replaced existing copyright notices with correct ones (the university owns the copyright of stuff developed by people working for the university or what students produce during their projects/theses)
```

- core/AdditionalMeasureProducer.html (1.8)
- core/AttributeExpression.html (1.4)
- core/AttributeStats.html (1.9)
- core/BinarySparseInstance.html (1.11)
- core/CheckOptionHandler.html (1.12)
- core/ContingencyTables.html (1.7)
- core/Copyable.html (1.6)
- core/Drawable.html (1.9)
- core/FastVector.html (1.14)

2007-05-01 01:24 fracpete

```
replaced existing copyright notices with correct ones (the university owns the copyright of stuff developed by people working for the university or what students produce during their projects/theses)
```

- classifiers/trees/j48/InfoGainSplitCrit.html (1.9)
- classifiers/trees/j48/ModelSelection.html (1.7)
- classifiers/trees/j48/NBTreeClassifierTree.html (1.5)
- classifiers/trees/j48/NBTreeModelSelection.html (1.4)
- classifiers/trees/j48/NBTreeNoSplit.html (1.3)
- classifiers/trees/j48/NBTreeSplit.html (1.4)
- classifiers/trees/j48/NoSplit.html (1.8)
- classifiers/trees/j48/PruneableClassifierTree.html (1.11)
- classifiers/trees/j48/SplitCriterion.html (1.7)
- classifiers/trees/j48/Stats.html (1.8)
- classifiers/trees/lmt/ResidualModelSelection.html (1.3)
- classifiers/trees/lmt/ResidualSplit.html (1.3)
- classifiers/trees/m5/CorrelationSplitInfo.html (1.3)
- classifiers/trees/m5/Impurity.html (1.7)
- classifiers/trees/m5/M5Base.html (1.18)
- classifiers/trees/m5/PreConstructedLinearModel.html (1.5)
- classifiers/trees/m5/Rule.html (1.14)
- classifiers/trees/m5/RuleNode.html (1.12)
- classifiers/trees/m5/SplitEvaluate.html (1.2)
- classifiers/trees/m5/Values.html (1.6)
- classifiers/trees/m5/YongSplitInfo.html (1.3)
- clusterers/Clusterer.html (1.16)
- clusterers/Cobweb.html (1.23)
- clusterers/DensityBasedClusterer.html (1.7)
- clusterers/FarthestFirst.html (1.8)
- clusterers/NumberOfClustersRequestable.html (1.2)
- clusterers/SimpleKMeans.html (1.29)

2007-05-01 01:24 fracpete

```
replaced existing copyright notices with correct ones (the university owns the copyright of stuff developed by people working for the university or what students produce during their projects/theses)
```

- datagenerators/Test.html (1.4)
- datagenerators/classifiers/classification/RDG1.html (1.4)
- datagenerators/clusterers/BIRCHCluster.html (1.7)
- datagenerators/clusterers/SubspaceCluster.html (1.4)
- estimators/CheckEstimator.html (1.3)
- estimators/ConditionalEstimator.html (1.6)
- estimators/DDConditionalEstimator.html (1.7)
- estimators/DKConditionalEstimator.html (1.7)
- estimators/DNConditionalEstimator.html (1.7)
- estimators/DiscreteEstimator.html (1.9)
- estimators/Estimator.html (1.9)
- estimators/EstimatorUtils.html (1.3)
- estimators/IncrementalEstimator.html (1.2)
- estimators/KDConditionalEstimator.html (1.7)
- estimators/KKConditionalEstimator.html (1.7)
- estimators/KernelEstimator.html (1.7)
- estimators/MahalanobisEstimator.html (1.7)
- estimators/NDConditionalEstimator.html (1.6)
- estimators/NNConditionalEstimator.html (1.7)
- estimators/NormalEstimator.html (1.8)
- estimators/PoissonEstimator.html (1.7)
- experiment/AveragingResultProducer.html (1.17)
- experiment/CSVResultListener.html (1.12)
- experiment/ClassifierSplitEvaluator.html (1.27)

2007-05-01 01:24 fracpete

```
replaced existing copyright notices with correct ones (the university owns the copyright of stuff developed by people working for the university or what students produce during their projects/theses)
```

- classifiers/CostMatrix.html (1.15)
- classifiers/IntervalEstimator.html (1.2)
- classifiers/IteratedSingleClassifierEnhancer.html (1.4)
- classifiers/IterativeClassifier.html (1.3)
- classifiers/MultipleClassifiersCombiner.html (1.6)
- classifiers/RandomizableClassifier.html (1.4)
- classifiers/RandomizableIteratedSingleClassifierEnhancer.html (1.4)
- classifiers/RandomizableMultipleClassifiersCombiner.html (1.4)
- classifiers/RandomizableSingleClassifierEnhancer.html (1.4)
- classifiers/SingleClassifierEnhancer.html (1.7)
- classifiers/Sourcable.html (1.5)
- classifiers/UpdateableClassifier.html (1.5)
- classifiers/bayes/BayesNet.html (1.30)

2007-05-01 01:24 fracpete

```
replaced existing copyright notices with correct ones (the university owns the copyright of stuff developed by people working for the university or what students produce during their projects/theses)
```

- filters/unsupervised/attribute/ChangeDateFormat.html (1.6)
- filters/unsupervised/attribute/Copy.html (1.6)
- filters/unsupervised/attribute/Discretize.html (1.12)
- filters/unsupervised/attribute/FirstOrder.html (1.8)
- filters/unsupervised/attribute/MakeIndicator.html (1.5)
- filters/unsupervised/attribute/MathExpression.html (1.8)
- filters/unsupervised/attribute/NominalToBinary.html (1.11)
- filters/unsupervised/attribute/Normalize.html (1.8)
- filters/unsupervised/attribute/NumericToBinary.html (1.7)
- filters/unsupervised/attribute/NumericTransform.html (1.8)
- filters/unsupervised/attribute/Obfuscate.html (1.6)
- filters/unsupervised/attribute/PKIDiscretize.html (1.8)
- filters/unsupervised/attribute/PotentialClassIgnorer.html (1.6)
- filters/unsupervised/attribute/Remove.html (1.8)
- filters/unsupervised/attribute/RemoveType.html (1.7)
- filters/unsupervised/attribute/RemoveUseless.html (1.9)
- filters/unsupervised/attribute/ReplaceMissingValues.html (1.8)
- filters/unsupervised/attribute/Standardize.html (1.8)
- filters/unsupervised/attribute/StringToNominal.html (1.8)
- filters/unsupervised/attribute/StringToWordVector.html (1.19)
- filters/unsupervised/attribute/SwapValues.html (1.8)
- filters/unsupervised/attribute/TimeSeriesDelta.html (1.6)

2007-05-01 01:24 fracpete

```
replaced existing copyright notices with correct ones (the university owns the copyright of stuff developed by people working for the university or what students produce during their projects/theses)
```

- classifiers/functions/SVMreg.html (1.3)
- classifiers/functions/SimpleLinearRegression.html (1.9)
- classifiers/functions/SimpleLogistic.html (1.12)
- classifiers/functions/VotedPerceptron.html (1.22)
- classifiers/functions/neural/LinearUnit.html (1.6)
- classifiers/functions/neural/NeuralConnection.html (1.6)
- classifiers/functions/neural/NeuralMethod.html (1.6)
- classifiers/functions/neural/NeuralNode.html (1.7)
- classifiers/functions/neural/SigmoidUnit.html (1.6)
- classifiers/functions/pace/ChisqMixture.html (1.4)
- classifiers/functions/pace/DiscreteFunction.html (1.3)
- classifiers/functions/pace/MixtureDistribution.html (1.4)
- classifiers/functions/pace/NormalMixture.html (1.4)
- classifiers/functions/pace/PaceMatrix.html (1.5)
- classifiers/functions/supportVector/Kernel.html (1.7)
- classifiers/functions/supportVector/NormalizedPolyKernel.html (1.6)
- classifiers/functions/supportVector/PolyKernel.html (1.6)
- classifiers/functions/supportVector/Puk.html (1.2)
- classifiers/functions/supportVector/RBFKernel.html (1.7)
- classifiers/functions/supportVector/RegOptimizer.html (1.2)
- classifiers/functions/supportVector/RegSMO.html (1.3)
- classifiers/functions/supportVector/RegSMOImproved.html (1.2)
- classifiers/functions/supportVector/SMOset.html (1.4)

2007-05-01 01:24 fracpete

```
replaced existing copyright notices with correct ones (the university owns the copyright of stuff developed by people working for the university or what students produce during their projects/theses)
```

- gui/arffviewer/ArffPanel.html (1.8)
- gui/arffviewer/ArffSortedTableModel.html (1.4)
- gui/arffviewer/ArffTable.html (1.8)
- gui/arffviewer/ArffTableCellRenderer.html (1.4)
- gui/arffviewer/ArffTableModel.html (1.8)
- gui/arffviewer/ArffViewer.html (1.8)
- gui/beans/AbstractDataSink.html (1.7)
- gui/beans/AbstractDataSinkBeanInfo.html (1.2)
- gui/beans/AbstractDataSource.html (1.4)
- gui/beans/AbstractDataSourceBeanInfo.html (1.3)
- gui/beans/AbstractEvaluator.html (1.5)
- gui/beans/AbstractTestSetProducer.html (1.5)
- gui/beans/AbstractTestSetProducerBeanInfo.html (1.2)
- gui/beans/AbstractTrainAndTestSetProducer.html (1.5)
- gui/beans/AbstractTrainAndTestSetProducerBeanInfo.html (1.2)
- gui/beans/AbstractTrainingSetProducer.html (1.6)

2007-05-01 01:24 fracpete

```
replaced existing copyright notices with correct ones (the university owns the copyright of stuff developed by people working for the university or what students produce during their projects/theses)
```

- gui/boundaryvisualizer/BoundaryPanelDistributed.html (1.7)
- gui/boundaryvisualizer/BoundaryVisualizer.html (1.19)
- gui/boundaryvisualizer/DataGenerator.html (1.4)
- gui/boundaryvisualizer/KDDataGenerator.html (1.7)
- gui/boundaryvisualizer/RemoteBoundaryVisualizerSubTask.html (1.4)
- gui/boundaryvisualizer/RemoteResult.html (1.3)
- gui/experiment/DatasetListPanel.html (1.25)
- gui/experiment/DistributeExperimentPanel.html (1.6)
- gui/experiment/Experimenter.html (1.12)
- gui/experiment/GeneratorPropertyIteratorPanel.html (1.10)
- gui/experiment/HostListPanel.html (1.5)
- gui/experiment/OutputFormatDialog.html (1.7)
- gui/experiment/ResultsPanel.html (1.42)
- gui/experiment/RunNumberPanel.html (1.6)
- gui/experiment/RunPanel.html (1.21)
- gui/experiment/SetupModePanel.html (1.5)
- gui/experiment/SetupPanel.html (1.31)
- gui/experiment/SimpleSetupPanel.html (1.14)
- gui/explorer/AssociationsPanel.html (1.26)
- gui/explorer/AttributeSelectionPanel.html (1.48)

2007-05-01 01:24 fracpete

```
replaced existing copyright notices with correct ones (the university owns the copyright of stuff developed by people working for the university or what students produce during their projects/theses)
```

- gui/visualize/LegendPanel.html (1.7)
- gui/visualize/MatrixPanel.html (1.18)
- gui/visualize/Plot2D.html (1.25)
- gui/visualize/Plot2DCompanion.html (1.5)
- gui/visualize/PlotData2D.html (1.18)
- gui/visualize/PostscriptGraphics.html (1.4)
- gui/visualize/PostscriptWriter.html (1.3)
- gui/visualize/PrintableComponent.html (1.7)
- gui/visualize/PrintableHandler.html (1.2)
- gui/visualize/PrintablePanel.html (1.3)
- gui/visualize/ThresholdVisualizePanel.html (1.5)
- gui/visualize/VisualizePanel.html (1.28)
- gui/visualize/VisualizePanelEvent.html (1.4)
- gui/visualize/VisualizePanelListener.html (1.5)
- gui/visualize/VisualizeUtils.html (1.8)

2007-05-01 01:24 fracpete

```
replaced existing copyright notices with correct ones (the university owns the copyright of stuff developed by people working for the university or what students produce during their projects/theses)
```

- attributeSelection/GreedyStepwise.html (1.9)
- attributeSelection/HoldOutSubsetEvaluator.html (1.6)
- attributeSelection/InfoGainAttributeEval.html (1.20)
- attributeSelection/OneRAttributeEval.html (1.19)
- attributeSelection/PrincipalComponents.html (1.35)
- attributeSelection/RandomSearch.html (1.17)
- attributeSelection/RankSearch.html (1.17)
- attributeSelection/RankedOutputSearch.html (1.11)
- attributeSelection/Ranker.html (1.25)
- attributeSelection/ReliefFAttributeEval.html (1.21)
- attributeSelection/SVMAttributeEval.html (1.26)
- attributeSelection/StartSetHandler.html (1.8)
- attributeSelection/SubsetEvaluator.html (1.10)
- attributeSelection/SymmetricalUncertAttributeEval.html (1.20)
- attributeSelection/UnsupervisedAttributeEvaluator.html (1.9)
- attributeSelection/UnsupervisedSubsetEvaluator.html (1.9)
- attributeSelection/WrapperSubsetEval.html (1.29)
- classifiers/BVDecompose.html (1.13)
- classifiers/CheckClassifier.html (1.32)
- classifiers/Classifier.html (1.17)

2007-05-01 01:24 fracpete

```
replaced existing copyright notices with correct ones (the university owns the copyright of stuff developed by people working for the university or what students produce during their projects/theses)
```

- classifiers/trees/Id3.html (1.20)
- classifiers/trees/J48.html (1.6)
- classifiers/trees/LMT.html (1.10)
- classifiers/trees/M5P.html (1.9)
- classifiers/trees/NBTree.html (1.9)
- classifiers/trees/REPTree.html (1.25)
- classifiers/trees/RandomForest.html (1.12)
- classifiers/trees/RandomTree.html (1.18)
- classifiers/trees/UserClassifier.html (1.27)
- classifiers/trees/adtree/PredictionNode.html (1.6)
- classifiers/trees/adtree/ReferenceInstances.html (1.4)
- classifiers/trees/adtree/Splitter.html (1.4)
- classifiers/trees/adtree/TwoWayNominalSplit.html (1.5)
- classifiers/trees/adtree/TwoWayNumericSplit.html (1.5)
- classifiers/trees/j48/BinC45ModelSelection.html (1.10)
- classifiers/trees/j48/BinC45Split.html (1.13)
- classifiers/trees/j48/C45ModelSelection.html (1.10)
- classifiers/trees/j48/C45PruneableClassifierTree.html (1.14)
- classifiers/trees/j48/C45Split.html (1.12)
- classifiers/trees/j48/ClassifierSplitModel.html (1.10)
- classifiers/trees/j48/ClassifierTree.html (1.21)
- classifiers/trees/j48/Distribution.html (1.11)
- classifiers/trees/j48/EntropyBasedSplitCrit.html (1.7)
- classifiers/trees/j48/EntropySplitCrit.html (1.7)
- classifiers/trees/j48/GainRatioSplitCrit.html (1.7)

2007-05-01 01:24 fracpete

```
replaced existing copyright notices with correct ones (the university owns the copyright of stuff developed by people working for the university or what students produce during their projects/theses)
```

- gui/beans/PredictionAppenderBeanInfo.html (1.3)
- gui/beans/PredictionAppenderCustomizer.html (1.3)
- gui/beans/SaverBeanInfo.html (1.2)
- gui/beans/SaverCustomizer.html (1.7)
- gui/beans/ScatterPlotMatrix.html (1.8)
- gui/beans/ScatterPlotMatrixBeanInfo.html (1.2)
- gui/beans/StartUpListener.html (1.2)
- gui/beans/StripChart.html (1.14)
- gui/beans/StripChartBeanInfo.html (1.2)
- gui/beans/StripChartCustomizer.html (1.4)
- gui/beans/TestSetEvent.html (1.4)
- gui/beans/TestSetListener.html (1.3)
- gui/beans/TestSetMaker.html (1.5)
- gui/beans/TestSetMakerBeanInfo.html (1.2)
- gui/beans/TestSetProducer.html (1.3)
- gui/beans/TextEvent.html (1.4)
- gui/beans/TextListener.html (1.3)
- gui/beans/TextViewer.html (1.11)
- gui/beans/TextViewerBeanInfo.html (1.2)
- gui/beans/ThresholdDataEvent.html (1.3)
- gui/beans/ThresholdDataListener.html (1.2)
- gui/beans/TrainTestSplitMaker.html (1.8)
- gui/beans/TrainTestSplitMakerBeanInfo.html (1.2)
- gui/beans/TrainTestSplitMakerCustomizer.html (1.4)
- gui/beans/TrainingSetEvent.html (1.4)
- gui/beans/TrainingSetListener.html (1.4)
- gui/beans/TrainingSetMaker.html (1.5)
- gui/beans/TrainingSetMakerBeanInfo.html (1.3)
- gui/beans/TrainingSetProducer.html (1.3)
- gui/beans/UserRequestAcceptor.html (1.3)
- gui/beans/Visible.html (1.2)
- gui/beans/VisualizableErrorEvent.html (1.3)
- gui/beans/VisualizableErrorListener.html (1.2)
- gui/beans/WekaWrapper.html (1.4)
- gui/boundaryvisualizer/BoundaryPanel.html (1.22)

2007-05-01 01:24 fracpete

```
replaced existing copyright notices with correct ones (the university owns the copyright of stuff developed by people working for the university or what students produce during their projects/theses)
```

- core/converters/AbstractSaver.html (1.4)
- core/converters/ArffSaver.html (1.6)
- core/converters/BatchConverter.html (1.2)
- core/converters/C45Saver.html (1.6)
- core/converters/CSVSaver.html (1.7)
- core/converters/DatabaseConnection.html (1.6)
- core/converters/DatabaseConverter.html (1.2)
- core/converters/DatabaseSaver.html (1.10)
- core/converters/FileSourcedConverter.html (1.4)
- core/converters/IncrementalConverter.html (1.2)
- core/converters/Saver.html (1.3)
- core/converters/SerializedInstancesSaver.html (1.6)
- core/converters/URLSourcedLoader.html (1.2)
- core/converters/XRFFSaver.html (1.3)
- core/matrix/DoubleVector.html (1.3)
- core/matrix/ExponentialFormat.html (1.3)
- core/matrix/FlexibleDecimalFormat.html (1.3)
- core/matrix/FloatingPointFormat.html (1.3)
- core/matrix/IntVector.html (1.3)
- core/stemmers/IteratedLovinsStemmer.html (1.6)
- core/stemmers/LovinsStemmer.html (1.6)
- datagenerators/ClassificationGenerator.html (1.4)
- datagenerators/ClusterGenerator.html (1.6)

2007-05-01 01:24 fracpete

```
replaced existing copyright notices with correct ones (the university owns the copyright of stuff developed by people working for the university or what students produce during their projects/theses)
```

- gui/graphvisualizer/LayoutCompleteEvent.html (1.5)
- gui/graphvisualizer/LayoutCompleteEventListener.html (1.5)
- gui/graphvisualizer/LayoutEngine.html (1.5)
- gui/streams/InstanceCounter.html (1.5)
- gui/streams/InstanceEvent.html (1.7)
- gui/streams/InstanceJoiner.html (1.6)
- gui/streams/InstanceListener.html (1.4)
- gui/streams/InstanceLoader.html (1.5)
- gui/streams/InstanceProducer.html (1.5)
- gui/streams/InstanceSavePanel.html (1.5)
- gui/streams/InstanceTable.html (1.5)
- gui/streams/InstanceViewer.html (1.5)
- gui/streams/SerialInstanceListener.html (1.4)
- gui/treevisualizer/Colors.html (1.5)
- gui/treevisualizer/Edge.html (1.4)
- gui/treevisualizer/NamedColor.html (1.4)
- gui/treevisualizer/Node.html (1.4)
- gui/treevisualizer/NodePlace.html (1.5)
- gui/treevisualizer/PlaceNode1.html (1.4)
- gui/treevisualizer/PlaceNode2.html (1.4)
- gui/treevisualizer/TreeBuild.html (1.4)
- gui/treevisualizer/TreeDisplayEvent.html (1.4)
- gui/treevisualizer/TreeDisplayListener.html (1.5)
- gui/treevisualizer/TreeVisualizer.html (1.13)
- gui/visualize/AttributePanel.html (1.11)
- gui/visualize/AttributePanelEvent.html (1.4)
- gui/visualize/AttributePanelListener.html (1.5)
- gui/visualize/ClassPanel.html (1.16)
- gui/visualize/JComponentWriter.html (1.4)
- gui/visualize/JPEGWriter.html (1.5)

2007-05-01 01:24 fracpete

```
replaced existing copyright notices with correct ones (the university owns the copyright of stuff developed by people working for the university or what students produce during their projects/theses)
```

- filters/StreamableFilter.html (1.2)
- filters/SupervisedFilter.html (1.2)
- filters/UnsupervisedFilter.html (1.2)
- filters/supervised/attribute/AttributeSelection.html (1.7)
- filters/supervised/attribute/ClassOrder.html (1.8)
- filters/supervised/attribute/Discretize.html (1.7)
- filters/supervised/attribute/NominalToBinary.html (1.7)
- filters/supervised/instance/SpreadSubsample.html (1.7)
- filters/supervised/instance/StratifiedRemoveFolds.html (1.6)
- filters/unsupervised/attribute/AbstractTimeSeries.html (1.8)
- filters/unsupervised/attribute/Add.html (1.6)
- filters/unsupervised/attribute/AddCluster.html (1.10)
- filters/unsupervised/attribute/AddExpression.html (1.7)

2007-05-01 01:24 fracpete

```
replaced existing copyright notices with correct ones (the university owns the copyright of stuff developed by people working for the university or what students produce during their projects/theses)
```

- associations/LabeledItemSet.html (1.4)
- associations/PriorEstimation.html (1.6)
- associations/RuleGeneration.html (1.3)
- associations/RuleItem.html (1.4)
- attributeSelection/ASEvaluation.html (1.14)
- attributeSelection/ASSearch.html (1.11)
- attributeSelection/AttributeEvaluator.html (1.10)
- attributeSelection/AttributeSelection.html (1.46)
- attributeSelection/AttributeTransformer.html (1.8)
- attributeSelection/BestFirst.html (1.27)
- attributeSelection/CfsSubsetEval.html (1.26)
- attributeSelection/ChiSquaredAttributeEval.html (1.14)
- attributeSelection/ClassifierSubsetEval.html (1.17)
- attributeSelection/ConsistencySubsetEval.html (1.15)
- attributeSelection/ErrorBasedMeritEvaluator.html (1.6)
- attributeSelection/ExhaustiveSearch.html (1.14)
- attributeSelection/GainRatioAttributeEval.html (1.21)
- attributeSelection/GeneticSearch.html (1.18)

2007-05-01 01:24 fracpete

```
replaced existing copyright notices with correct ones (the university owns the copyright of stuff developed by people working for the university or what students produce during their projects/theses)
```

- classifiers/bayes/net/search/local/GeneticSearch.html (1.4)
- classifiers/bayes/net/search/local/HillClimber.html (1.8)
- classifiers/bayes/net/search/local/K2.html (1.6)
- classifiers/bayes/net/search/local/RepeatedHillClimber.html (1.5)
- classifiers/bayes/net/search/local/SimulatedAnnealing.html (1.4)
- classifiers/bayes/net/search/local/TAN.html (1.6)
- classifiers/bayes/net/search/local/TabuSearch.html (1.4)
- classifiers/evaluation/ConfusionMatrix.html (1.8)
- classifiers/evaluation/CostCurve.html (1.8)
- classifiers/evaluation/EvaluationUtils.html (1.10)
- classifiers/evaluation/MarginCurve.html (1.10)
- classifiers/evaluation/NominalPrediction.html (1.11)
- classifiers/evaluation/NumericPrediction.html (1.8)
- classifiers/evaluation/Prediction.html (1.7)
- classifiers/evaluation/ThresholdCurve.html (1.21)
- classifiers/evaluation/TwoClassStats.html (1.8)
- classifiers/functions/IsotonicRegression.html (1.6)
- classifiers/functions/LinearRegression.html (1.23)
- classifiers/functions/Logistic.html (1.37)
- classifiers/functions/MultilayerPerceptron.html (1.11)
- classifiers/functions/PaceRegression.html (1.8)
- classifiers/functions/RBFNetwork.html (1.9)
- classifiers/functions/SMO.html (1.64)

2007-05-01 01:24 fracpete

```
replaced existing copyright notices with correct ones (the university owns the copyright of stuff developed by people working for the university or what students produce during their projects/theses)
```

- gui/InstancesSummaryPanel.html (1.6)
- gui/JTableHelper.html (1.2)
- gui/ListSelectorDialog.html (1.9)
- gui/Loader.html (1.2)
- gui/LogPanel.html (1.17)
- gui/Logger.html (1.5)
- gui/LookAndFeel.html (1.3)
- gui/PropertyDialog.html (1.7)
- gui/PropertyPanel.html (1.13)
- gui/PropertySelectorDialog.html (1.9)
- gui/PropertySheetPanel.html (1.17)
- gui/PropertyText.html (1.8)
- gui/PropertyValueSelector.html (1.7)
- gui/ResultHistoryPanel.html (1.25)
- gui/SaveBuffer.html (1.7)
- gui/SelectedTagEditor.html (1.8)
- gui/SetInstancesPanel.html (1.13)
- gui/SimpleCLI.html (1.10)
- gui/SimpleCLIPanel.html (1.4)
- gui/SysErrLog.html (1.4)
- gui/TaskLogger.html (1.5)
- gui/ViewerDialog.html (1.4)
- gui/WekaTaskMonitor.html (1.8)

2007-05-01 01:24 fracpete

```
replaced existing copyright notices with correct ones (the university owns the copyright of stuff developed by people working for the university or what students produce during their projects/theses)
```

- classifiers/meta/nestedDichotomies/ClassBalancedND.html (1.7)
- classifiers/meta/nestedDichotomies/DataNearBalancedND.html (1.7)
- classifiers/meta/nestedDichotomies/ND.html (1.8)
- classifiers/misc/HyperPipes.html (1.20)
- classifiers/misc/VFI.html (1.14)
- classifiers/misc/monotone/InstancesUtil.html (1.2)
- classifiers/rules/ConjunctiveRule.html (1.15)
- classifiers/rules/DecisionTable.html (1.42)
- classifiers/rules/DecisionTableHashKey.html (1.2)
- classifiers/rules/JRip.html (1.21)
- classifiers/rules/M5Rules.html (1.10)
- classifiers/rules/OneR.html (1.23)
- classifiers/rules/PART.html (1.8)
- classifiers/rules/Prism.html (1.22)
- classifiers/rules/Ridor.html (1.19)
- classifiers/rules/Rule.html (1.7)
- classifiers/rules/RuleStats.html (1.6)
- classifiers/rules/ZeroR.html (1.15)
- classifiers/rules/part/C45PruneableDecList.html (1.8)
- classifiers/rules/part/ClassifierDecList.html (1.12)
- classifiers/rules/part/MakeDecList.html (1.16)
- classifiers/rules/part/PruneableDecList.html (1.9)
- classifiers/trees/ADTree.html (1.7)
- classifiers/trees/DecisionStump.html (1.23)

2007-05-01 01:24 fracpete

```
replaced existing copyright notices with correct ones (the university owns the copyright of stuff developed by people working for the university or what students produce during their projects/theses)
```

- gui/beans/ConnectionNotificationConsumer.html (1.2)
- gui/beans/CrossValidationFoldMaker.html (1.9)
- gui/beans/CrossValidationFoldMakerBeanInfo.html (1.2)
- gui/beans/CrossValidationFoldMakerCustomizer.html (1.3)
- gui/beans/CustomizerCloseRequester.html (1.2)
- gui/beans/CustomizerClosingListener.html (1.4)
- gui/beans/DataSetEvent.html (1.4)
- gui/beans/DataSink.html (1.2)
- gui/beans/DataSource.html (1.2)
- gui/beans/DataSourceListener.html (1.3)
- gui/beans/DataVisualizer.html (1.11)
- gui/beans/DataVisualizerBeanInfo.html (1.2)
- gui/beans/EventConstraints.html (1.3)
- gui/beans/Filter.html (1.16)
- gui/beans/FilterBeanInfo.html (1.4)
- gui/beans/FilterCustomizer.html (1.9)
- gui/beans/GraphEvent.html (1.5)
- gui/beans/GraphListener.html (1.4)
- gui/beans/GraphViewer.html (1.8)
- gui/beans/GraphViewerBeanInfo.html (1.2)
- gui/beans/IncrementalClassifierEvaluator.html (1.11)
- gui/beans/IncrementalClassifierEvaluatorBeanInfo.html (1.3)
- gui/beans/IncrementalClassifierEvent.html (1.9)
- gui/beans/IncrementalClassifierListener.html (1.3)
- gui/beans/InstanceEvent.html (1.5)
- gui/beans/InstanceListener.html (1.3)
- gui/beans/KnowledgeFlow.html (1.29)
- gui/beans/KnowledgeFlowApp.html (1.18)
- gui/beans/LoaderBeanInfo.html (1.2)
- gui/beans/LoaderCustomizer.html (1.14)
- gui/beans/LogWriter.html (1.2)
- gui/beans/MetaBean.html (1.9)
- gui/beans/ModelPerformanceChart.html (1.7)
- gui/beans/ModelPerformanceChartBeanInfo.html (1.2)
- gui/beans/PredictionAppender.html (1.15)

2007-05-01 01:24 fracpete

```
replaced existing copyright notices with correct ones (the university owns the copyright of stuff developed by people working for the university or what students produce during their projects/theses)
```

- core/Matchable.html (1.6)
- core/MathematicalExpression.html (1.3)
- core/Matrix.html (1.24)
- core/NoSupportForMissingValuesException.html (1.3)
- core/Optimization.html (1.8)
- core/Option.html (1.6)
- core/OptionHandler.html (1.8)
- core/ProtectedProperties.html (1.5)
- core/Queue.html (1.9)
- core/RandomVariates.html (1.4)
- core/Randomizable.html (1.3)
- core/Range.html (1.17)
- core/SelectedTag.html (1.10)
- core/SerializedObject.html (1.10)
- core/SingleIndex.html (1.7)
- core/SpecialFunctions.html (1.7)
- core/Stopwords.html (1.5)
- core/Summarizable.html (1.6)
- core/SystemInfo.html (1.4)
- core/Tag.html (1.9)
- core/UnassignedClassException.html (1.5)
- core/UnassignedDatasetException.html (1.5)
- core/Undoable.html (1.2)
- core/UnsupportedAttributeTypeException.html (1.5)
- core/UnsupportedClassTypeException.html (1.5)
- core/WeightedInstancesHandler.html (1.5)
- core/WekaException.html (1.5)
- core/converters/AbstractFileSaver.html (1.4)

2007-05-01 01:24 fracpete

```
replaced existing copyright notices with correct ones (the university owns the copyright of stuff developed by people working for the university or what students produce during their projects/theses)
```

- gui/explorer/ClustererPanel.html (1.60)
- gui/explorer/Explorer.html (1.42)
- gui/explorer/PreprocessPanel.html (1.71)
- gui/graphvisualizer/BIFFormatException.html (1.5)
- gui/graphvisualizer/BIFParser.html (1.7)
- gui/graphvisualizer/DotParser.html (1.4)
- gui/graphvisualizer/GraphConstants.html (1.5)
- gui/graphvisualizer/GraphEdge.html (1.4)
- gui/graphvisualizer/GraphNode.html (1.4)
- gui/graphvisualizer/GraphVisualizer.html (1.7)
- gui/graphvisualizer/HierarchicalBCEngine.html (1.5)

2007-04-30 22:23 fracpete

```
runTokenize(Tokenizer,String[]) and tokenize(Tokenizer,String[]) now read from stdin if there is no data supplied in the options array. useful if one wants to use the tokenizers in pipes
```

- core/tokenizers/Tokenizer.html (1.2)

2007-04-29 23:15 fracpete

```
added "updateFinished()" method to UpdateableClusterer interface for signalling that all instances have been added. can be used for some clean-up operations, calculations, etc.
```

- clusterers/UpdateableClusterer.html (1.2)

2007-04-29 23:09 fracpete

```
initial import of contribution of an n-gram tokenizer by Sebastian Germesin
```

- core/tokenizers/NGramTokenizer.html (1.1)

2007-04-29 23:06 fracpete

```
initial import of Trie data structure
```

- core/Trie.html (1.1)

2007-04-29 23:05 fracpete

```
added cache for find(Class,String) method for faster retrieval of class names
added findPackages() method to obtain all the available package names in the classpath
updated main method for either outputting packages or classes
```

- core/ClassDiscovery.html (1.5)

2007-04-25 22:19 fracpete

```
initial import
decoupled the tokenizers from the StringToWordVector
```

- core/tokenizers/AlphabeticTokenizer.html (1.1)
- core/tokenizers/WordTokenizer.html (1.1)

2007-04-23 20:29 fracpete

```
Vote is now derived from RandomizableMultipleClassifiersCombiner in order to provide repeatable results: distributionForInstanceMajorityVoting was using Math.random() to break ties instead of a seeded java.util.Random object
```

- classifiers/meta/Vote.html (1.14)

2007-04-23 08:01 fracpete

```
main method now outputs initial and max memory
```

- core/Memory.html (1.6)

2007-04-22 21:58 fracpete

```
method batchFinished now processes data only if there's data available
```

- filters/SimpleBatchFilter.html (1.9)

2007-04-22 07:38 fracpete

```
fixed bug in batch mode: didn't check for m_FirstBatchDone flag and therefore buffered instances in second batch again instead of processing and returning them immediately (thanks to Juan Jose Rodriguez)
```

- filters/SimpleStreamFilter.html (1.8)

2007-04-19 23:59 fracpete

```
fixed listOptions and Javadoc regarding number of sub-committees (it's 3 not 10)
thanks to Juan Jose Rodriguez
```

- classifiers/meta/MultiBoostAB.html (1.15)

2007-04-16 22:24 fracpete

```
fixed bug in instanceToArray method: was using the wrong index
thanks to Jesus Maudes and Juan Jose Rodriguez for their extensive tests
https://list.scms.waikato.ac.nz/mailman/htdig/wekalist/2007-April/009671.html
```

- classifiers/functions/LibSVM.html (1.9)

2007-04-12 21:03 fracpete

```
saveToFile and loadFromFile now use the weka.core.SerializationHelper
```

- core/Debug.html (1.7)

2007-03-29 02:24 fracpete

```
the user can now specify width and height of the image that is generated, this removes the limitation of not being able to get all of the component into the image (cut off, since only a screenshot)
```

- gui/visualize/BMPWriter.html (1.2)
- gui/visualize/PNGWriter.html (1.3)

2007-03-28 21:39 fracpete

```
added possibility to specify capabilities a scheme should NOT have, e.g., one can now search for schemes that support binary classes but not nominal classes (i.e., more than 2 class labels)
```

- core/FindWithCapabilities.html (1.3)

2007-03-25 21:13 fracpete

```
initial import of MS SQL Server 2005 Express Edition setup
```

- experiment/DatabaseUtils.props.mssqlserver2005 (1.2)

2007-03-23 04:52 fracpete

```
SQL Viewer saves/restores the panel size
```

- gui/sql/InfoPanel.html (1.3)
- gui/sql/ResultPanel.html (1.3)
- gui/sql/SqlViewer.html (1.5)
- gui/sql/SqlViewerDialog.html (1.3)

2007-03-20 03:24 fracpete

```
corrected copyright
```

- classifiers/bayes/NaiveBayesMultinomial.html (1.15)

2007-03-13 04:27 fracpete

```
attribute and relation names are now also converted into valid content, otherwise a single/double quote in the name would generate invalid XML code
```

- core/xml/XMLInstances.html (1.3)

2007-03-12 20:58 fracpete

```
fixed bug in evaluate(Kernel,Instances) method: the kernel's eval method was called with the indices swapped
the equals method now checks for NaNs (2 NaNs are now considered to be equal)
```

- classifiers/functions/supportVector/KernelEvaluation.html (1.2)

2007-02-21 02:42 fracpete

```
fixed Javadoc
```

- filters/unsupervised/attribute/NominalToString.html (1.2)

2007-02-21 01:07 fracpete

```
classifier is defaulting back to ZeroR model if data contains only the class attribute
```

- classifiers/bayes/WAODE.html (1.2)

2007-02-15 00:41 fracpete

```
for centralized copyright information (dialogs, logs, etc.)
```

- core/Copyright.html (1.1)
- core/Copyright.props (1.1)

2007-02-13 01:54 fracpete

```
introduced new type for STRING attributes: TEXT = 9 (one couldn't load columns as STRING attributes, they were always converted into NOMINAL ones)
```

- experiment/DatabaseUtils.props (1.7)
- experiment/DatabaseUtils.props.hsql (1.4)
- experiment/DatabaseUtils.props.mssqlserver (1.3)
- experiment/DatabaseUtils.props.mysql (1.3)
- experiment/DatabaseUtils.props.odbc (1.4)
- experiment/DatabaseUtils.props.oracle (1.3)
- experiment/DatabaseUtils.props.postgresql (1.3)

2007-02-13 00:50 fracpete

```
added warning about cross-validation to globalInfo (and therefore the Javadoc)
```

- classifiers/misc/SerializedClassifier.html (1.2)

2007-02-11 20:40 fracpete

```
fixed setOptions method (flag interpretation was broken - GUI was working fine)
added "-remove-old-class" flag: if used with -classification and a serialized claassifier one can batch-classify files
```

- filters/supervised/attribute/AddClassification.html (1.2)

2007-02-11 20:33 fracpete

```
updated Javadoc, "-silent" option was missing
```

- core/GlobalInfoJavadoc.html (1.6)
- core/OptionHandlerJavadoc.html (1.7)
- core/TechnicalInformationHandlerJavadoc.html (1.5)

2007-02-11 20:33 fracpete

```
updated Javadoc, "-silent" option was missing
```

- core/AllJavadoc.html (1.4)

2007-02-08 22:47 fracpete

```
Explorer tabs are now arbitrary plugins (only the PreprocessPanel is fixed):
classes derived from JPanel and implementing at least weka.gui.explorer.Explorer.ExplorerPanel
more information on the Wiki:
http://weka.sourceforge.net/wiki/index.php/Adding_tabs_in_the_Explorer
```

- gui/explorer/Explorer.props (1.5)
- gui/explorer/ExplorerDefaults.html (1.3)
- gui/explorer/VisualizePanel.html (1.15)

2007-02-08 02:19 fracpete

```
enabled selection of directories (e.g., for recursive dataset selection in the Experimenter)
fixed bug with the "All Files" filter (was always enabled)
```

- gui/ConverterFileChooser.html (1.7)

2007-02-07 04:26 fracpete

```
added serialVersionUID (packages weka.associations and weka.attributeSelection got already checked in on 05/02/2007 before the repository was tagged)
```

- filters/SimpleFilter.html (1.4)
- gui/CheckBoxList.html (1.2)
- gui/LogWindow.html (1.4)
- gui/SimpleDateFormatEditor.html (1.2)
- gui/SplashWindow.html (1.2)

2007-02-07 04:26 fracpete

```
added serialVersionUID (packages weka.associations and weka.attributeSelection got already checked in on 05/02/2007 before the repository was tagged)
```

- core/matrix/QRDecomposition.html (1.3)
- core/matrix/SingularValueDecomposition.html (1.3)

2007-02-07 04:26 fracpete

```
added serialVersionUID (packages weka.associations and weka.attributeSelection got already checked in on 05/02/2007 before the repository was tagged)
```

- associations/tertius/SimpleLinkedList.html (1.5)
- classifiers/functions/supportVector/CachedKernel.html (1.7)
- classifiers/lazy/LBR.html (1.11)
- classifiers/lazy/kstar/KStarCache.html (1.9)
- classifiers/meta/EnsembleSelection.html (1.3)

2007-02-07 04:26 fracpete

```
added serialVersionUID (packages weka.associations and weka.attributeSelection got already checked in on 05/02/2007 before the repository was tagged)
```

- gui/ensembleLibraryEditor/AddModelsPanel.html (1.2)
- gui/ensembleLibraryEditor/DefaultModelsPanel.html (1.2)
- gui/ensembleLibraryEditor/ListModelsPanel.html (1.2)
- gui/ensembleLibraryEditor/LoadModelsPanel.html (1.2)
- gui/explorer/DataGeneratorPanel.html (1.4)
- gui/sql/DbUtils.html (1.2)
- gui/sql/InfoPanelCellRenderer.html (1.2)
- gui/sql/QueryPanel.html (1.3)
- gui/sql/ResultSetTable.html (1.2)
- gui/sql/ResultSetTableCellRenderer.html (1.2)
- gui/sql/event/ConnectionEvent.html (1.2)
- gui/sql/event/HistoryChangedEvent.html (1.2)
- gui/sql/event/QueryExecuteEvent.html (1.2)
- gui/sql/event/ResultChangedEvent.html (1.2)

2007-02-07 04:26 fracpete

```
added serialVersionUID (packages weka.associations and weka.attributeSelection got already checked in on 05/02/2007 before the repository was tagged)
```

- datagenerators/RegressionGenerator.html (1.3)
- experiment/ResultMatrix.html (1.8)
- experiment/ResultMatrixCSV.html (1.3)
- experiment/ResultMatrixGnuPlot.html (1.4)
- experiment/ResultMatrixHTML.html (1.3)
- experiment/ResultMatrixLatex.html (1.5)
- experiment/ResultMatrixPlainText.html (1.7)
- experiment/ResultMatrixSignificance.html (1.4)

2007-02-07 04:26 fracpete

```
added serialVersionUID (packages weka.associations and weka.attributeSelection got already checked in on 05/02/2007 before the repository was tagged)
```

- clusterers/RandomizableClusterer.html (1.2)
- clusterers/RandomizableDensityBasedClusterer.html (1.2)
- clusterers/RandomizableSingleClustererEnhancer.html (1.2)
- clusterers/SingleClustererEnhancer.html (1.3)
- clusterers/forOPTICSAndDBScan/DataObjects/EuclidianDataObject.html (1.3)
- clusterers/forOPTICSAndDBScan/DataObjects/ManhattanDataObject.html (1.3)
- clusterers/forOPTICSAndDBScan/Databases/SequentialDatabase.html (1.3)
- clusterers/forOPTICSAndDBScan/OPTICS\_GUI/GraphPanel.html (1.3)
- clusterers/forOPTICSAndDBScan/OPTICS\_GUI/ResultVectorTableModel.html (1.3)
- clusterers/forOPTICSAndDBScan/OPTICS\_GUI/SERObject.html (1.3)
- core/InstanceComparator.html (1.3)
- core/matrix/CholeskyDecomposition.html (1.3)
- core/matrix/EigenvalueDecomposition.html (1.3)
- core/matrix/LUDecomposition.html (1.3)
- core/matrix/Matrix.html (1.7)

2007-02-06 22:42 fracpete

```
added test for serialVersionUID
```

- attributeSelection/CheckAttributeSelection.html (1.5)
- classifiers/functions/supportVector/CheckKernel.html (1.2)
- clusterers/CheckClusterer.html (1.8)

2007-02-06 22:41 fracpete

```
helper class for dealing with serialVersionUIDs and serialization (reading and writing to and fro files/streams)
```

- core/SerializationHelper.html (1.1)

2007-02-04 21:07 fracpete

```
filters now return their Capabilities based on the data: in case there's no class attribute set, they'll remove all the types of classes that are supported apart from NO_CLASS, and in case that there's a class attribute present, then the NO_CLASS capability will be removed
Wavelet filter supports now also nominal attributes (restriction to numeric class was unnecessary)
AddCluster/ClusterMembership always have to ignore the class attribute for the capabilities because of the clusterers they're using internally
```

- filters/unsupervised/attribute/Wavelet.html (1.3)

2007-01-31 03:43 fracpete

```
added serialVersionUID
```

- attributeSelection/AttributeSetEvaluator.html (1.3)

2007-01-31 02:35 fracpete

```
added serialVersionUID
```

- associations/tertius/AttributeValueLiteral.html (1.5)
- associations/tertius/Body.html (1.5)
- associations/tertius/Head.html (1.5)
- associations/tertius/IndividualInstance.html (1.4)
- associations/tertius/IndividualInstances.html (1.5)
- associations/tertius/IndividualLiteral.html (1.4)
- associations/tertius/Literal.html (1.4)
- associations/tertius/LiteralSet.html (1.6)
- associations/tertius/Predicate.html (1.4)
- associations/tertius/Rule.html (1.6)
